# Targeting microenvironment in cancer therapeutics

**DOI:** 10.18632/oncotarget.9824

**Published:** 2016-06-05

**Authors:** Matthew Martin, Han Wei, Tao Lu

**Affiliations:** ^1^ Department of Pharmacology and Toxicology, Indiana University School of Medicine, Indianapolis, IN, USA; ^2^ Department of Biochemistry and Molecular Biology, Indiana University School of Medicine, Indianapolis, IN, USA; ^3^ Department of Medical and Molecular Genetics, Indiana University School of Medicine, Indianapolis, IN, USA

**Keywords:** cancer, microenvironment, cytokine, transcription factor

## Abstract

During development of a novel treatment for cancer patients, the tumor microenvironment and its interaction with the tumor cells must be considered. Aspects such as the extracellular matrix (ECM), the epithelial-mesenchymal transition (EMT), secreted factors, cancer-associated fibroblasts (CAFs), the host immune response, and tumor-associated microphages (TAM) are critical for cancer progression and metastasis. Additionally, signaling pathways such as the nuclear factor κB (NF-κB), transforming growth factor β (TGFβ), and tumor necrosis factor α (TNFα) can promote further cytokine release in the tumor environment, and impact tumor progression greatly. Importantly, cytokine overexpression has been linked to drug resistance in cancers and is therefore an attractive target for combinational therapies. Specific inhibitors of cytokines involved in signaling between tumor cells and the microenvironment have not been studied in depth and have great potential for use in personalized medicines. Together, the interactions between the microenvironment and tumors are critical for tumor growth and promotion and should be taken into serious consideration for future novel therapeutic approaches.

## INTRODUCTION

The cells surrounding a tumor make up a molecular microenvironment known as the stroma. The stroma can be influenced and can in turn influence the growth and formation of tumors and new metastases. Origination of this microenvironment can be linked to the “seed and soil” concept with the original cancer cells, termed “seeds,” and the microenvironment, termed “soil.” The cancer cells grow in new metastases just as “seeds” can grow in new areas of “soil” [1]. Like soil in nature, the components in the microenvironment are very complex. It includes the extracellular matrix (ECM), a variety of secreted factors, as well as different types of cells.

ECM plays a critical role in the tumor microenvironment. In order to form a tumor, cancer cells must form attachments to the ECM and communicate with other cells, such as tumor-associated neutrophils, cancer-associated fibroblasts, and myeloid-derived suppressor cells. Then, the tumor cells can colonize the microenvironment and form a metastasis [2]. The ECM is especially important in tumor formation and invasion as cells respond and adapt to the local microenvironment to progress to malignancy. This involves both deregulated proliferation of tumor cells and modification of the immediate environment to favor cell survival, angiogenesis, and spread of the tumor. Evidence has shown that aberrant expression of factors that regulate the ECM can lead to increased tumor formation. For instance, decrease of secreted protein acidic and rich in cysteine (SPARC), a protein involved in regulation of morphogenesis and cellular differentiation, led to increase of tumor formation of implanted tumors [3]. Inhibition of chemokines such as growth-regulated α protein (GROα, also named CXCL1) can also increase cell proliferation in melanoma, leading to enhanced tumor growth [4]. Moreover, overexpression of cytokines such as tumor necrosis factor α (TNFα), a cytokine involved in several signaling pathways and in inflammation, could lead to increased tumor formation in nude mice [5]. Factors that can degrade the ECM also facilitate tumor formation and invasion. For instance, matrix metalloproteinases (MMPs) have been implicated in degradation of basement membranes and ECM, facilitating tumor cell invasion and metastasis as well as promoting cell proliferation, survival, and angiogenesis [6].

Besides the ECM, another important aspect that can lead to cancer formation and invasion is a process termed epithelial-mesenchymal transition (EMT) of cancer cells. EMT can increase cancer cells’ migratory capacity, invasiveness, resistance to apoptosis, and the production of ECM components [7]. Many factors originating in the microenvironment can help to induce EMT, including epidermal growth factor (EGF), platelet-derived growth factor (PDGF), and transforming growth factor β (TGFβ). These factors may functionally activate EMT-inducing transcription factors, such as Snail (SNAI1), Slug (SNAI2), zinc finger E-box binding homeobox 1 (ZEB1), Twist family BHLH Transcription Factor 1 (Twist), Goosecoid (GSC), and forkhead box protein C2 (FOXC2) in cancer cells []. For example, TGFβ has been implicated in the repression of e-cadherin, leading to the increased invasiveness of tumor cells and the activation of Sma and Mad (SMAD) and mitogen-activated protein kinase (MAPK) signaling pathways, which all can contribute to the induction of EMT [14]In addition to different cytokines and transcription factors, disruption of cell-cell adhesion molecules such as cadherins and the cell-ECM adhesions mediated by integrins can also contribute to EMT, resulting in tumor invasion [15, 16].

“Invasion” of cancer and “metastasis formation” involve the steps of *intravasation* (the invasion of cancer cells through the basal membrane into a blood or lymphatic vessel), *extravasation* (transport through the circulation*)*, formation of *micrometastases*, and *colonization* [8, 15]. Both “Invasion” and “metastatic formation” require a specific microenvironment. “Invasion” is affected by the microenvironment in many ways. Activation of nearby cells in the stroma including fibroblasts, endothelial cells and macrophages can lead to reorganization of the ECM and release of growth factors, therefore promoting tumor growth [17–19]. On the other hand, even though “metastatic formation” is very heterogeneous and adaptive to diverse molecular and unhospitable microenvironments, it can still be facilitated by signaling of cells and other factors of the cancer microenvironment [20]. Macrophages in the microenvironment can promote invasion of cancer cells by producing EGF. Tumor-associated macrophages (TAMs) gather around blood vessels and create gradients of EGF within the tumor environment that attract tumor cells towards blood vessels and promote intravasation [21].

In addition to ECM, EMT, and the different factors described above, non-cancerous cells also comprise a major part of the tumor environment that can signal and be signaled by released chemokines and cytokines. For instance, the work of Luis Parada showed that mice with neurofibromatosis type 1 with only a loss of*Nf1*heterozygosity in Schwann cells were unable to form neurofibroma, but that*Nf1*haploinsufficiency in a second type of cell - mast cells, was required for tumor formation. A separate cell-signaling ligand, stem cell growth factor (SGF), which binds to cKIT, a SCF receptor, was also needed for mast cell recruitment for tumor formation [22].

In short, the “soil” made up of the proteins, growth factors, and other non-tumor cells is a crucial part of tumor formation, invasion, and metastasis. It is important to consider and understand the interplay between the microenvironment and tumors when pursuing future therapeutics for cancers. Below, we will discuss some aspects of this microenvironment in details.

## CYTOKINE SECRETION, INFLAMMATION, THE HOST IMMUNE RESPONSE AND TUMOR PROGRESSION

A major part of the tumor microenvironment are different secreted factors, which play important roles in inflammation and cell growth. For many years, inflammation has been known to play a role in tumor formation. In 1991, Parsonnet *et al.* reported that after infecting cells with bacteria, they observed an increased development of cancer where there was inflammation [23]. They confirmed that constant cell proliferation in the stroma of a tumor was associated with an environment rich in inflammatory cells [23].

The relationship between inflammation and cancer is deeply intertwined. Inflammation is induced in tumor through neoplasia-associated angiogenesis and lymphangiogenesis. This creates an organization of blood vessels and lymphatics where neoplastic cells can interact with other mesenchymal, hematopoietic, lymphoid cells, and the ECM. Importantly, neoplastic cells themselves can produce arrays of cytokines and chemokines that are chemoattractants for other non-cancer cells, such as granulocytes, mast cells, monocytes/macrophages, fibroblasts and endothelial cells, all of which are needed for tumor formation. Besides cytokine release, proteolytic enzymes such as metalloproteases can also be secreted through activated fibroblasts and infiltrating inflammatory cells. Together these released factors can coordinate and act to recruit cells to the location of tumorigenesis by remodeling of the ECM. Ultimately, these factors promote tumor growth, stimulate angiogenesis, induce fibroblast migration and maturation, and enable metastatic spread through invasion into other systems.

To date, ample evidence suggests that TAMs aid in inflammation-mediated infiltration of neoplastic tumors as well. TAMs can be activated by interleukin-2 (IL-2) to go through apoptosis, but also can produce many angiogenic and lymphogenic growth factors which further mediate neoplastic progression [24]. Fusion of TAMs and normal cells has also been found as a way for the tumor cells and microenvironment to communicate in a cell-cell manner [25]. Chronic overexpression of mediators of inflammation, such as TNFα andTGFβ produced by TAMs, have been identified in different types of cancers [26, 27]. Moreover, cancer stem cells (CSC) are also mediated by cytokines released by TAMs and can be selected through overexpression of cytokines identified through gene arrays [28]. In addition to TAM-mediated cytokine release, recruitment of inflammatory cells to the tumor microenvironment is also influenced by hypoxic conditions in the tumor. For instance, hypoxic conditions cause hypoxia-sensitive genes to be expressed in tumor cells, resulting in the recruitment of inflammatory cells such as macrophages and granulocytes to the tumor microenvironment [29, 30], which will in turn contribute to the production of reactive oxygen species (ROS), leading to the activation of the nuclear factor κB (NF-κB)pathway and subsequent secretion of TNFα and other pro-inflammatory cytokines to promote cell proliferation [31].

The host immune response to a tumor greatly impacts the tumor microenvironment and the latter can in turn attenuate the immune response. These processes are extremely interconnected. In particular, tumor cells and the tumor microenvironment can contribute to the evasion and suppression of the host immune response. Inflammatory cells infiltrate tumors as part of the immune response to slow tumor progression. This process is known as immune surveillance [32]. The immune cells infiltrating the tumor contain cells from both the adaptive and innate immune responses. Adaptive immune response cells include tumor-infiltrating lymphocytes (TILs), dendritic cells, and B cells, while innate immune response cells include macrophages, polymorphonuclear leukocytes and a few natural killer cells [33]. Of these, TILs are the major component of immune infiltrates in tumors. These cells are affected greatly by antigens released in the tumor microenvironment, resulting in decreased lymphocyte proliferation, decreased signaling through T cell receptors, and reduced ability to mediate cytotoxicity of tumor targets. This collective immune suppressive effort for TILs allows the tumor to escape destruction by immune systems [34, 35].

Interestingly, besides the tumor itself and the tumor environment, one type of T cell, namely the regulatory T cells (Tregs), also contribute greatly to the immune suppressive effect. Tregs accumulate and expand in the tumor microenvironment and produce excessive interleukin 10 (IL-10) and TGFβ1. Contact-dependent interactions of IL-10 and TGFβ1 with T cells lead to decreased proliferation of immune cells and prevent the adaptive immune response. On the other hand, increased levels of TGFβ1 also promotes tumor cell proliferation, leading to increased tumor formation. In addition to the above functions, increased levels of Tregs also interfere with the function of anti-tumor effector cells known as cytotoxic T lymphocytes (CTLs), preventing their function of signaling the destruction of tumor cells [36].

Collectively, the above evidence suggests complex interactions between tumor cells, tumor environment, and the smart escape of tumor cells from the host immune responses.

## CANCER ASSOCIATED FIBROBLASTS (CAFS)

Researchers have long believed that tumors are “wounds that do not heal” [37] CAFs are activated fibroblasts that share similarities with fibroblasts, including the deposition of ECM. The tumor microenvironment, including epithelial differentiation, inflammation, and wound healing [38, 39] promotes tumor growth, angiogenesis, inflammation, and metastasis by various mechanisms [40]. Different cellular origins and tumor-derived factors shape the phenotype of CAFs and contribute to their appearance as a heterogeneous cell population with distinct subtypes. The activation factor of CAFs might vary. Several activation factors have been frequently reported in the stromal fibroblasts of solid tumors, including α-smooth muscle actin (α-SMA), fibroblast-specific protein, PDGF receptors-β and fibroblast activation protein (FAP) [41–43].

The role of CAFs in tumor progression is multi-faceted as they can either inhibit or promote malignant growth [44]. In the early stages of tumor progression, CAFs function as repressors by facilitating the formation of gap junctions between activated fibroblasts and leading to contact inhibition on cancer cells. However, at a later stage, CAFs become activated by several tumor-secreted factors, such as TGFβ1, PDGFα/β, basic fibroblast growth factor (b-FGF), or interleukin 6 (IL-6) [44], resulting in the promotion of both tumor growth and progression [44]. However, what exactly may cause the CAFs’ transition from “tumor suppressor” to “tumor promoter” during tumor progress is still not completely clear. Recently, Liu lab reported that the activity of Notch1 signaling pathway negatively regulates tumor growth phenotype in CAFs [45]. In a skin melanoma mouse model, Liu's group reported that CAF carrying elevated Notch1 activity significantly inhibited melanoma growth and invasion, while CAFs with null Notch1 promoted melanoma invasion [45]. The role of CAFs in cancer progression expands a wide range, including ECM remodeling, secretion of soluble factors, regulation of motility and stemness, tumor metabolism remodeling, and preparation of metastatic niche. CAFs affect tumor cell proliferation through growth factors, hormones, and cytokines [44]. For instance, CAFs from hepatocellular carcinoma (HCC) (H-CAFs) promoted the growth of HCC and correlated positively with tumor size. The concentration of hepatocyte growth factor (HGF) secreted in the conditioned medium of H-CAFs was higher than that from normal skin fibroblasts (NSFs). Importantly, anti-HGF significantly reduced the proliferation-promoting capability of H-CAFs [46]. This example inarguably supports the promoting role of H-CAFs related microenvironment in HCC tumor growth and therapy.

## TRANSCRIPTION FACTORS AND MICROENVIRONMENT

Researchers recently noticed that the transcription factor, the NF-κB plays a central role to link the inflammatory state and colon cancer. In addition to NF-κB stimulating the proliferation of tumor cells and enhancing their survival through the regulation of anti-apoptotic genes [47], an important function of NF-κB is regulating the expression of various cytokines and growth factors which contribute the tumor promoting microenvironment, resulting in tumor progression. Interestingly, different types of post-translational modifications can regulate NF-κB activity, including methylation, phosphorylation, ubiquitination, and acetylation. Recent evidence from our laboratory indicates that NF-κB can be phosphorylated at multiple sites (S316, S529 and S536) in response to interleukin 1β (IL-1β) treatment. These three sites either individually or cooperatively, regulated the secretion of distinct groups of NF-κB-dependent cytokines and growth factors, leading to an autocrine loop that in turn further activated NF-κB and promoter colon cancer progression [48].

Besides the posttranslational modification of NF-κB itself, novel activators of NF-κB, such as the transcription factor Y-box protein 1 (YBX1) is highly expressed in many cancers and is an excellent marker for cancer.YBX1 was recently investigated by our lab, and e found that overexpression of YBX1 is connected to activation ofNF-κB. This leads to the secretion of a group of pro-inflammatory cytokines such as TNFα andTGFβ, which in turn activates the expression of genes encoding other inflammatory cytokines, cell adhesion molecules, and cell survival proteins. This expression leads to the accelerated colon cancer progression [49]. We further found that YBX1 can be activated through phosphorylation on its S165 residue. Mutation of S165-A (alanine) on YBX1 led to decreased cytokine and chemokine secretion, as well as reduced cell growth and tumorigenic ability in colon cancer cells [49].

## TUMOR MICROENVIRONMENT INDUCES DRUG RESISTANCE

The cytokines in the microenvironment not only can contribute to tumor growth, invasion, and metastasis, but in some situations they also drive the development of drug resistance. Resistance of anti-cancer drugs is a major cause of treatment failure in cancer. Recent studies on therapeutic resistance mechanisms have been conferred largely by alterations not in the tumor cells, but in their environment, indicating the importance of understanding the tumor cell heterogeneity [50]. For instance, in healthy tissue, the stroma functions as the main barrier against tumorigenesis. However, in the presence of the tumor, the stroma converts the environment to support cancer progression. Researchers noticed that the microenvironment could affect tumor vasculature, which serves as a barrier to optimal drug delivery [51]. A study showed that cytoreduction (referring to reducing the number of cancer cells) of the stroma through enzymatic destruction of hyaluronan, could reduce interstitial pressure and improve vessel patency and flow. This would lead to drug delivery improvement, as indicated by the increased efficacy of standard-of-care chemotherapy when combined with hyaluronan depletion in an animal model of pancreatic cancer (PC) [52]. To date, scientists have identified some critical molecular signaling pathways that are involved in the soluble factor-mediated drug resistance. One example involves stromal cell-derived factor 1 (SDF1) and its membrane-bound receptor, chemokine (C-X-C motif) receptor 4 (CXCR4). In chronic lymphocytic leukemia (CLL) cells, SDF1 produced by stromal cells can interact with CXCR4 and activate extracellular signal-regulated kinase 1/2 (ERK1/2) and Akt (also known as protein kinase B (PKB)) pathways, leading to anti-apoptotic responses and promoting tumor cell survival [53, 54]. Moreover, SDF1-mediated CXCR4 activation induces resistance to chemotherapy drug Cytarabine, by downregulating microRNA let-7a and promoting transcriptional activation of Myc and BCL2-like 1 isoform 1 (BCL-xL) in acute myeloid leukemia (AML) cells [55]. In human colon cancer, CXCR4 is overexpressed in the chemoresistant tumor cells. Stromal cells from lymph nodes promote resistance to 5-fluorouracil and oxaliplatin through a SDF1/CXCR4 dependent mechanism [56].

## PERSPECTIVES

### Targeting cytokines or growth factors therapeutically

Instead of a single tumor cell, it could be more beneficial to specifically target the pro-tumorigenic factors supplied by innate immune cells during chronic inflammation, such as treatment of colitis-associated colon cancer (CAC). Colon cancer is one of the leading causes of cancer-related mortality in US. The limited success achieved by only targeting tumor cells highlights the importance of understanding the role of the tumor microenvironment and its precise contribution to carcinogenesis. Inflammation, especially chronic inflammation, is a critical factor for colon tumor microenvironment. Clinical studies have proven that long-term use of nonsteroidal anti-inflammatory drugs by targeting cyclooxygenase-2 (COX-2) reduces the risk of colon cancer by 40-50% [57]. Furthermore, colitis or inflammatory bowel disease (IBD) is a key predisposing factor for colon cancer [47].

Though inhibition of cytokines or growth factors holds great therapeutic potentials, there are few federal Food and Drug Administration (FDA) approved examples. A wonderful clinical example is the application of Sultuximab, an anti-IL-6 in the treatment of smoldering multiple myeloma [58]. Additionally, Bevacizumab, an effective anti-vascular endothelial growth factor (VEGF) antibody, has been successfully used in colon cancer treatment [59]. The underdevelopment of anti-cytokine or growth factors therapeutics potentially opens up more possibilities for the development of new therapeutics along this line. Furthermore, use of a cytokine inhibitor and cytokine receptor inhibitor in a combinatorial therapy could potentially prove more effective than a single therapeutic.

**Figure 1:**
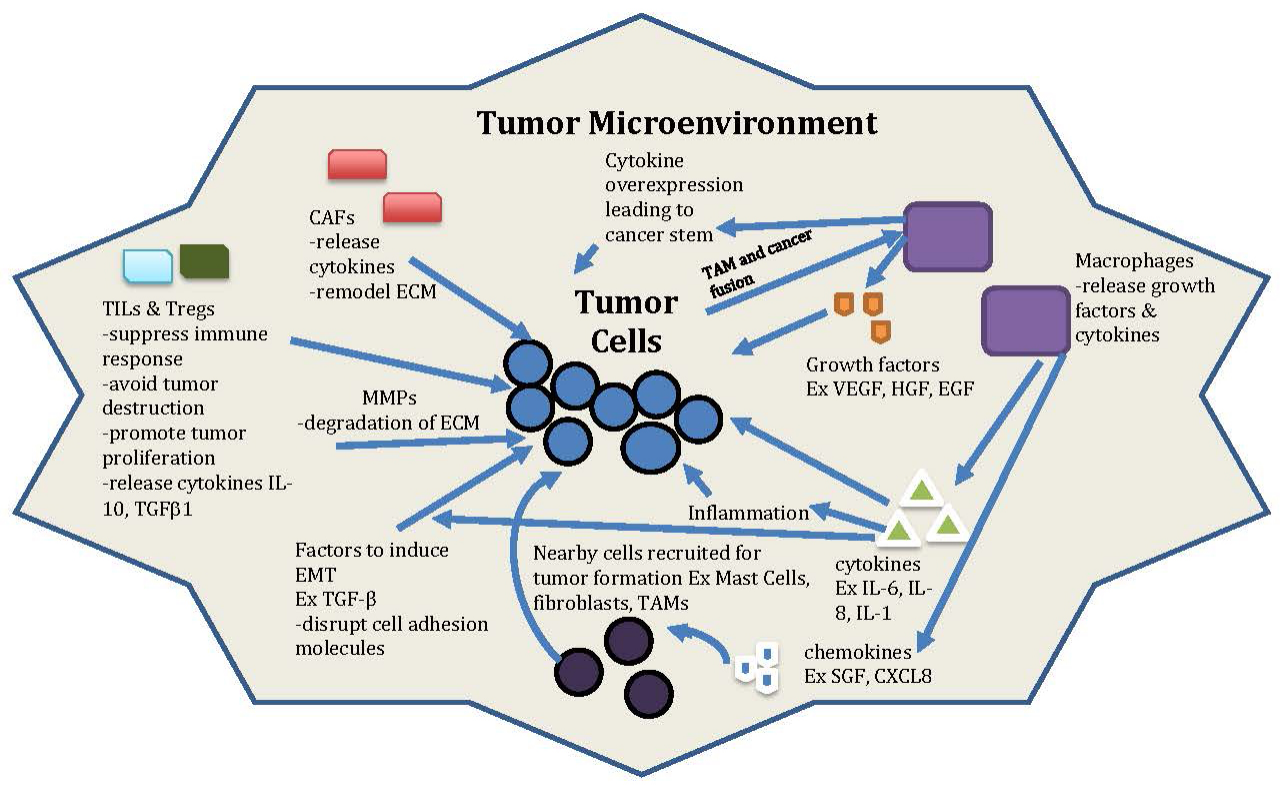
General model of tumor and microenvironment interactions Non cancer cells (such as CAFs, TAMs, Tregs, TILs), secreted factors (such as cytokines, chemokines, growth factors *etc.*), proteins (such as MMPs), transcription factors, and the ECM and EMT process are all involved in the interaction between tumor microenvironment with tumor cells. This complicated interaction can be the novel target for anti-cancer therapy in the future.

### Targeting the host immune system therapeutically

As we described above, since immune evasion by cancer cells is important for the progression of tumors [60, 61], when considering cancer therapeutics, it is important to think about the strategy of re-activating the immune system to recognize and kill tumor cells.

For instance, Sipuleucel-T, manufactured by the Dendreon Corporation, is a cell-based cancer immunotherapy for prostate cancer. It consists of peripheral blood mononuclear cells (PBMCs) that are fused with a prostatic acid phosphatase (PAP). Sipuleucel-T acts to stimulate antitumor T-cell response in prostate tumor cells containing PAP [62]. Another immunotherapeutic is ipilimumab, which is a monoclonal antibody that works to activate the immune system by targeting CTLA-4, a protein receptor that downregulates the immune responses of CTLs. Ipilimumab turns off this inhibitory mechanism by CTLA -4, allowing CTLs to function [62].

A recent hot topic in therapeutics for the immune system is the programmed death ligand 1 (PD-L1)/programmed death 1 (PD1) axis. PD1 is an immune inhibitory receptor expressed on several immune cells, particularly CTLs. The interaction of PD-L1 with PD-1 inhibits T-cell activation and cytokine production. In normal tissue, the PD-L1/PD1 ligation is critically important in maintaining homeostasis of immune response to prevent autoimmunity during infection or inflammation. However, in the tumor microenvironment, their interaction could turn off the CTLs and in turn lead to the immune escape of tumor cells. Consequently, disruption of the PD-L1/PD1 axis by inhibitors will boost the immune response against cancer. Recently, many therapeutics targeting the PD-L1/PD-1 axis were used in Phase I, II, and III clinical trials for a variety of cancers. These include nivolumab, pembrolizumab, which are PD-1 inhibitors, and durvalumab, avelumab as well as atezolizumab, which are PD-L1 inhibitors. All of these target the PD-L1/PD1 axis [63]. The response rate of patients to the therapeutics were highly variable, ranging from 80% with pembrolizumab in advanced Merkel cell carcinoma (MCC), a rare type of skin cancer, to 12% with durvalumab in recurrent/metastatic squamous cell carcinoma of head and neck (SCCHN). While some therapeutics were not very effective in clinical trials, almost all tested therapeutic approaches were a single therapeutic as opposed to combinatorial therapies. To date, few combinatorial therapies involving PD-L1/PD1 axis have been tried. Theoretically, combinations of PD-1 or PD-L1 inhibitors with other therapeutic approaches could potentially provide a more effective therapeutic strategy for patients. It won't be a huge surprise to see more exciting work coming out along this line of research in the near future.

### Challenges ahead

Despite the success in targeting non-tumor cell compartments in the past, significant challenges still lie ahead for implementing stromal targeting strategies in clinical practice. These range from difficulties in assessing the composition of the stroma in human tumors to correctly modeling pre-clinically the vast heterogeneity observed in human tumors and correlating this heterogeneity with outcome, drug response, and drug resistance [50]. For example, PC is an aggressive malignant disease with a 5-year survival rate of less than 5%. PC is one of the few cancers for which survival has not been improved substantially over nearly 40 years mainly due to limited success of target therapies. One reason for failure of systemic therapies is related to the abundant tumor stromal contents in the tumor microenvironment. The tumor microenvironment is constantly changing in composition. PC stroma is very heterogeneous and comprised of cellular components, such as fibroblasts, myofibroblasts, pancreatic stellate cells, immune cells, blood vessels, ECM, and soluble proteins such as cytokines and growth factors. PC stroma supports tumor growth and promotes metastasis and simultaneously serves as a physical barrier to drug delivery [64]. Understanding and altering stromal composition or function will definitely benefit the patients.

An additional consideration is the potential off-target effect. In the case of cytokines such as TGFβ or TNFα, the ubiquitousness of the cytokines may result in unforeseen off-target effects when targeting them with therapeutics [65]. For instance in Feyen *et al.*'s work TNFα inhibitors were not selective simply for TNFα, but could target many other proteins leading to unforeseen complications for patients. Since many cytokines are involved in several different signaling pathways such as the NF-κB pathway and MAPK pathways, targeting the cytokine itself can cause issues as it will change signaling in pathways not intended to be targeted. To be truly selective in our targeting of cancer cells, we must develop and use therapeutics that are more specific to what is involved between a tumor microenvironment and the tumor itself, but not normal cells. Additionally, changes in the tumor microenvironment can contribute to development of resistance, preventing rejection of the tumor by the immune system. Changes in the balance of cytokines such as VEGF, TGFβ, IL-4, IL-12 and others can actively suppress dendritic cell (DC) maturation which dampens the antigen presenting function of DCs, which then contributes to immune tolerance [66]. These are all the factors that we must consider in terms of developing novel anti-microenvironment therapies.

## References

[1] Mathot L, Stenninger J (2012). Behavior of seeds and soil in the mechanism of metastasis: a deeper understanding. Cancer Sci.

[2] Nguyen DX, Bos PD, Massagué J (2009). Metastasis: from dissemination to organ-specific colonization. Nat Rev Cancer.

[3] Brekken RA, Puolakkainen P, Graves DC, Workman G, Lubkin SR, Sage EH (2003). Enhanced growth of tumors in SPARC null mice is associated with changes in the ECM. J Clin Invest.

[4] Norgauer J, Metzner B, Schraufstätter I (1996). Expression and growth-promoting function of the IL-8 receptor beta in human melanoma cells. J Immun.

[5] Tracey K, Lowry S (1990). The role of cytokine mediators in septic shock. Adv Surg.

[6] Chambers AF, Matrisian LM (1997). Changing views of the role of matrix metalloproteinases in metastasis. J National Cancer Inst.

[7] Kalluri R, Neilson EG (2003). Epithelial-mesenchymal transition and its implications for fibrosis. J Clin Invest.

[8] Thiery JP (2002). Epithelial-mesenchymal transitions in tumour progression. Nat Rev Cancer.

[9] Jechlinger M, Grünert S, Beug H (2002). Mechanisms in epithelial plasticity and metastasis: insights from 3D cultures and expression profiling. J Mamm Gland Biol Neoplasia.

[10] Shi Y, Massagué J (2003). Mechanisms of TGF signaling from cell membrane to the nucleus. Cell.

[11] Niessen K, Fu Y, Chang L, Hoodless PA, McFadden D, Karsan A (2008). Slug is a direct Notch target required for initiation of cardiac cushion cellularization. J Cell Biol.

[12] Medici D, Hay ED, Olsen BR (2008). Snail and Slug promote epithelial-mesenchymal transition through -catenin-T-cell factor-4-dependent expression of transforming growth factor-3. Mol Biol Cell.

[13] Kokudo T, Suzuki Y, Yoshimatsu Y, Yamazaki T, Watabe T, Miyazono K (2008). Snail is required for TGF-induced endothelial-mesenchymal transition of embryonic stem cell-derived endothelial cells. J Cell Sci.

[14] Oft M, Heider K-H, Beug H (1998). TGF signaling is necessary for carcinoma cell invasiveness and metastasis. Curr Biol.

[15] Yang J, Weinberg RA (2008). Epithelial-mesenchymal transition: at the crossroads of development and tumor metastasis. Dev Cell.

[16] Weinberg RA (2008). Twisted epithelial-mesenchymal transition blocks senescence. Nat Cell Biol.

[17] Egeblad M, Rasch MG, Weaver VM (2010). Dynamic interplay between the collagen scaffold and tumor evolution. Curr Opin Cell Biol.

[18] Yao H, Zeng Z-Z, Fay KS, Veine DM, Staszewski ED, Morgan M, Wilder-Romans K, Williams TM, Spalding AC, Ben-Josef E (2011). Role of 5 1 integrin up-regulation in radiation-induced invasion by human pancreatic cancer cells. Transl Oncol.

[19] Nelson CM, Khauv D, Bissell MJ, Radisky DC (2008). Change in cell shape is required for matrix metalloproteinase-induced epithelial-mesenchymal transition of mammary epithelial cells. J Cell Biochem.

[20] Friedl P, Wolf K (2003). Tumour-cell invasion and migration: diversity and escape mechanisms. Nat Rev Cancer.

[21] Sahai E (2007). Illuminating the metastatic process. Nat Rev Cancer.

[22] Swartz MA, Iida N, Roberts EW, Sangaletti S, Wong MH, Yull FE, Coussens LM, DeClerck YA (2012). Tumor microenvironment complexity: emerging roles in cancer therapy. Cancer Res.

[23] Parsonnet J, Friedman GD, Vandersteen DP, Chang Y, Vogelman JH, Orentreich N, Sibley RK (1991). Helicobacter pylori infection and the risk of gastric carcinoma. New Eng J Med.

[24] Schoppmann SF, Birner P, Stöckl J, Kalt R, Ullrich R, Caucig C, Kriehuber E, Nagy K, Alitalo K, Kerjaschki D (2002). Tumor-associated macrophages express lymphatic endothelial growth factors and are related to peritumoral lymphangiogenesis. Am J Pathol.

[25] Powell AE, Anderson EC, Davies PS, Silk AD, Pelz C, Impey S, Wong MH (2011). Fusion between Intestinal epithelial cells and macrophages in a cancer context results in nuclear reprogramming. Cancer Res.

[26] Moustakas A, Pardali K, Gaal A, Heldin C-H (2002). Mechanisms of TGF signaling in regulation of cell growth and differentiation. Immunol Lett.

[27] Torisu H, Ono M, Kiryu H, Furue M, Ohmoto Y, Nakayama J, Nishioka Y, Sone S, Kuwano M (2000). Macrophage infiltration correlates with tumor stage and angiogenesis in human malignant melanoma: Possible involvement of TNF and IL-1. Int J Cancer.

[28] Birnie R, Bryce SD, Roome C, Dussupt V, Droop A, Lang SH, Berry PA, Hyde CF, Lewis JL, Stower MJ (2008). Gene expression profiling of human prostate cancer stem cells reveals a pro-inflammatory phenotype and the importance of extracellular matrix interactions. Genome Biol.

[29] Denko NC, Fontana LA, Hudson KM, Sutphin PD, Raychaudhuri S, Altman R (2003). Investigating hypoxic tumor physiology through gene expression patterns. Oncogene.

[30] Aller MA, Arias JL, Nava MP, Arias J (2004). Posttraumatic inflammation is a complex response based on the pathological expression of the nervous, immune and endocrine function systems. Exp Biol Med.

[31] Lluis JM, Buricchi F, Chiarugi P, Morales A, Fernandez-Checa JC (2007). Dual role of mitochondrial reactive oxygen species in hypoxia signaling: activation of nuclear factor-kappa B via cSRC and oxidant dependent cell death. Cancer Res.

[32] Zitvogel L, Tesniere A, Kroemer G (2006). Cancer despite immunosurveillance: immunoselection and immunosubversion. Nat Rev Immunol.

[33] Whiteside TL, Kaufmann H, Wolchok JD (2007). The Local Tumor Microenvironment. General Principles of Tumor Immunotherapy: Basic and Clinical Applications of Tumor Immunology.

[34] Uzzo RG, Clark PE, Rayman P, Bloom T, Rybicki L, Novick AC, Bukowski RM, Finke JH (1999). Alterations in NFkappaB activation in T lymphocytes of patients with renal cell carcinoma. J. Nat. Cancer Inst.

[35] Kiessling R, Kono K, Petersson M, Wasserman K (1996). Immunosuppression in human tumor-host interaction: role of cytokines and alterations in signal-transducing molecules. Springer Sem. Immunopathol.

[36] Ferrone S, Whiteside TL (2007). Tumor microenvironment and immune escape. Surg. Oncol. Clinics N. Amer.

[37] Dvorak HF (1986). Tumors: wounds that do not heal: similarities between tumor stroma generation and wound healing. New Eng J Med.

[38] Chang HY, Sneddon JB, Alizadeh AA, Sood R, West RB, Montgomery K, Chi J-T, Van De Rijn M, Botstein D, Brown PO (2004). Gene expression signature of fibroblast serum response predicts human cancer progression: similarities between tumors and wounds. PLoS Biol.

[39] Cirri P, Chiarugi P (2011). Cancer associated fibroblasts: the dark side of the coin. Am J Cancer Res.

[40] Madar S, Goldstein I, Rotter V (2013). ‘Cancer associated fibroblasts’-more than meets the eye. Trends Mol Med.

[41] Kalluri R, Zeisberg M (2006). Fibroblasts in cancer. Nat Rev Cancer.

[42] Micke P (2004). Tumour-stroma interaction: cancer-associated fibroblasts as novel targets in anti-cancer therapy?. Lung Cancer.

[43] O'Brien P, O'Connor BF (2008). Seprase: an overview of an important matrix serine protease. Biochim Biophy Acta.

[44] Cirri P, Chiarugi P (2012). Cancer-associated-fibroblasts and tumour cells: a diabolic liaison driving cancer progression. Cancer Metastasis Rev.

[45] Shao H, Kong R, Ferrari ML, Radtke F, Capobianco AJ, Liu ZJ (2015). Notch1 Pathway Activity Determines the Regulatory Role of Cancer-Associated Fibroblasts in Melanoma Growth and Invasion. PLoS One.

[46] Jia C-C, Wang T-T, Liu W, Fu B-S, Hua X, Wang G-Y, Li T-J, Li X, Wu X-Y, Tai Y (2013). Cancer-associated fibroblasts from hepatocellular carcinoma promote malignant cell proliferation by HGF secretion. PLoS One.

[47] Schottelius AJ, Dinter H (2006). Cytokines, NF-B, microenvironment, intestinal inflammation and cancer. The Link Between Inflammation and Cancer.

[48] Wang B, Wei H, Prabhu L, Zhao W, Martin M, Hartley A-V, Lu T (2015). Role of Novel Serine 316 Phosphorylation of the p65 Subunit of NF-B in Differential Gene Regulation. J Biol Chem.

[49] Prabhu L, Mundade R, Wang B, Wei H, Hartley A-V, Martin M, McElyea K, Temm CJ, Sandusky G, Liu Y (2015). Critical role of phosphorylation of serine 165 of YBX1 on the activation of NF-B in colon cancer. Oncotarget.

[50] Junttila MR, de Sauvage FJ (2013). Influence of tumour micro-environment heterogeneity on therapeutic response. Nature.

[51] Jain RK (2005). Normalization of tumor vasculature: an emerging concept in antiangiogenic therapy. Science.

[52] Provenzano PP, Cuevas C, Chang AE, Goel VK, Von Hoff DD, Hingorani SR (2012). Enzymatic targeting of the stroma ablates physical barriers to treatment of pancreatic ductal adenocarcinoma. Cancer Cell.

[53] Burger JA, Tsukada N, Burger M, Zvaifler NJ, Dell'Aquila M, Kipps TJ (2000). Blood-derived nurse-like cells protect chronic lymphocytic leukemia B cells from spontaneous apoptosis through stromal cell-derived factor-1. Blood.

[54] Nishio M, Endo T, Tsukada N, Ohata J, Kitada S, Reed JC, Zvaifler NJ, Kipps TJ (2005). Nurselike cells express BAFF and APRIL, which can promote survival of chronic lymphocytic leukemia cells via a paracrine pathway distinct from that of SDF-1. Blood.

[55] Chen Y, Hui H, Yang H, Zhao K, Qin Y, Gu C, Wang X, Lu N, Guo Q (2013). Wogonoside induces cell cycle arrest and differentiation by affecting expression and subcellular localization of PLSCR1 in AML cells. Blood.

[56] Margolin DA, Silinsky J, Grimes C, Spencer N, Aycock M, Green H, Cordova J, Davis NK, Driscoll T, Li L (2011). Lymph node stromal cells enhance drug-resistant colon cancer cell tumor formation through SDF-1/CXCR4 paracrine signaling. Neoplasia.

[57] Smalley WE, DuBois RN (1997). Anti-inflammatory Drugs. Adv Pharm.

[58] Kurzrock R, Voorhees PM, Casper C, Furman RR, Fayad L, Lonial S, Borghaei H, Jagannath S, Sokol L, Usmani SZ (2013). A Phase I, Open-Label Study of Siltuximab, an Anti-IL-6 Monoclonal Antibody, in Patients with B-cell Non-Hodgkin Lymphoma, Multiple Myeloma, or Castleman Disease. Clin Cancer Res.

[59] Johnson DH, Fehrenbacher L, Novotny WF, Herbst RS, Nemunaitis JJ, Jablons DM, Langer CJ, DeVore RF, Gaudreault J, Damico LA (2004). Randomized phase II trial comparing bevacizumab plus carboplatin and paclitaxel with carboplatin and paclitaxel alone in previously untreated locally advanced or metastatic non-small-cell lung cancer. J Clin Oncol.

[60] Kaplan DH, Shankaran V, Dighe AS, Stockert E, Aguet M, Old LJ, Schreiber RD (1998). Demonstration of an interferon -dependent tumor surveillance system in immunocompetent mice. Proc Natl Acad Sci.

[61] Shankaran V, Ikeda H, Bruce AT, White JM, Swanson PE, Old LJ, Schreiber RD (2001). IFN and lymphocytes prevent primary tumor development and shape tumor immunogenicity. Nature.

[62] Sharma P, Wagner K, Wolchok JD, Allison JP (2011). Novel cancer immunotherapy agents with survival benefit: recent successes and next steps. Nat Rev Cancer.

[63] Kourie HR, Awada G, Awada AH (2016). Learning from the “tsunami” of immune checkpoint inhibitors in 2015. Critical Rev Oncol/Hematol.

[64] Feig C, Gopinathan A, Neesse A, Chan DS, Cook N, Tuveson DA (2012). The pancreas cancer microenvironment. Clin Cancer Res.

[65] Feyen O, Lueking A, Kowald A, Stephan C, Meyer HE, Göbel U, Niehues T (2008). Off-target activity of TNF- inhibitors characterized by protein biochips. Anal Bioanal Chem.

[66] Zou W (2005). Immunosuppressive networks in the tumour environment and their therapeutic relevance. Nat Rev Cancer.

